# Identification and Biological Evaluation of Secondary Metabolites from Marine Derived Fungi-*Aspergillus* sp. SCSIOW3, Cultivated in the Presence of Epigenetic Modifying Agents

**DOI:** 10.3390/molecules22081302

**Published:** 2017-08-04

**Authors:** Xiaofan Li, Zhenyao Xia, Jianqiang Tang, Jiahui Wu, Jing Tong, Mengjie Li, Jianhua Ju, Huirong Chen, Liyan Wang

**Affiliations:** 1 Shenzhen Key Laboratory of Marine Bioresource and Eco-environmental Science, College of Life Sciences and Oceanography, Shenzhen University, Shenzhen 518060, China; lixiaof@szu.edu.cn (X.L.); 18680378772@163.com (Z.X.); tangjianqiang888@163.com (J.T.); Hanne5@163.com (J.W.); 13689570230@163.com(J.T.); lmengjie16@163.com (M.L.); chenhr@szu.edu.cn (H.C.); 2 Shenzhen Key Laboratory of Microbial Genetic Engineering, College of Life Sciences and Oceanography, Shenzhen University, Shenzhen 518060, China; 3 CAS Key Laboratory of Tropical Marine Bio-resources and Ecology, Guangdong Key Laboratory of Marine Materia Medica, RNAM Center for Marine Microbiology, South China Sea Institute of Oceanology, Chinese Academy of Sciences, 164 West Xingang Road, Guangzhou 510301, China; jju@scsio.ac.cn

**Keywords:** marine fungus, chemical epigenetic modification, diphenyl ether, oxidative stress, algicidal activity

## Abstract

Chemical epigenetic manipulation was applied to a deep marine-derived fungus, *Aspergillus* sp. SCSIOW3, resulting in significant changes of the secondary metabolites. One new diphenylether-*O*-glycoside (diorcinol 3-*O*-α-D-ribofuranoside), along with seven known compounds, were isolated from the culture treated with a combination of histone deacetylase inhibitor (suberohydroxamic acid) and DNA methyltransferase inhibitor (5-azacytidine). Compounds **2** and **4** exhibited significant biomembrane protective effect of erythrocytes. **2** also showed algicidal activity against *Chattonella marina*, a bloom forming alga responsible for large scale fish deaths.

## 1. Introduction

With the recent completion of fungal genomes, it has become clear that the number of gene clusters encoding secondary metabolites greatly outnumbers the characterized compounds from these organisms [1]. It has been demonstrated that chemical epigenetic method is an effective technique for promoting the transcription of silent biosynthetic pathways and is well suited for the generation of structurally unique secondary metabolites [2,3,4]. Several different types of commercially available chemical epigenetic agents have been explored by several laboratories and provided valuable outcomes, including Zn^2+^ type histone deacetylase (HDAC) inhibitors: suberoylanilidehydroxamic acid (SAHA) and suberohydroxamic acid (SBHA); NAD type HDAC inhibitors: sirtinol, splitomycin, nicotinamide, and 2-anilino-benzamide; and DNA methyltransferase (DNMT) inhibitors: RG-108, procaine, and 5-azacytidine (5-AZA) [5]. Moreover, studies on the effect of the concomitant addition of these inhibitors have been conducted. A marked increase was observed in the secondary metabolite produced by *Isariatenuipes* and *Gibellulaformosana* cultivated in the presence of both SBHA and RG108 [6,7]. In order to maximize the opportunity for detecting novel secondary metabolites, we have begun using chemical epigenetic induction as a routine part of our screening program involving the exploration of marine derived fungi. Previously, we successfully obtained four new eremophilane-type sesquiterpenes from a deep marine derived fungi, *Aspergillus* sp. SCSIOW2, in the presence of a combination of SBHA and 5-AZA [8].

**Figure 1:**
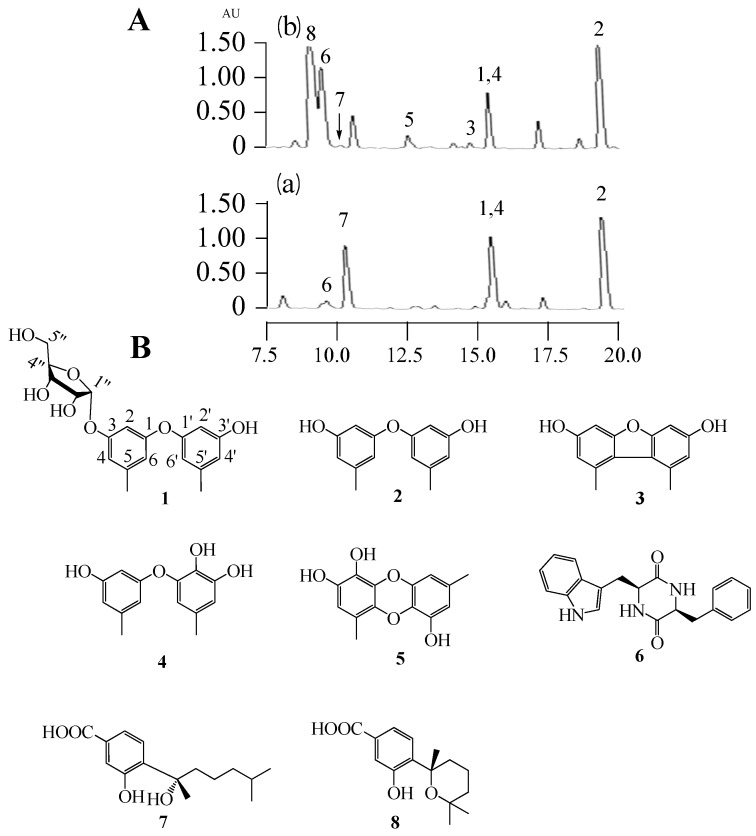
The secondary metabolite profile of *Aspergillus* sp. SCSIOW3 upon treatment with SBHA and 5-Aza. (**A**) HPLC-UV chromatogram at 254 nm of EtOAc extracts of untreated (**a**) and SBHA 1 mM + 5-Aza 1 mM treated (**b**) fermentation broth; (**B**) Structures of the identified substances **1**–**8**.

In this study, we applied a test bed of 72 marine derived fungi (strain numbers and properties shown in Table S1, Supporting Information) to assess changes in their biosynthetic products induced by adding a combination of 1 mM SBHA and 1 mM 5-AZA. The crude ethyl acetate extracts (from broths + or − SBHA and 5-AZA) were profiled by analytical RP C18 high pressure liquid chromatography (HPLC) and thin layer chromatography (TLC) to observe differences between the matched pairs. One member of this set, *Aspergillus* sp. SCSIOW3, showed noteworthy changes in the accumulation of some new peaks in HPLC (3, 5 and 8 were new emerging peaks; the amount of 6 was increased, 7 was decreased, and 1, 2 and 4 were not significantly changed. Figure 1A) and was chosen for further study. Therefore, the EtOAc extract of this culture was scaled-up and separated by using column chromatography and semi-preparative HPLC, resulting in the isolation of one new compound, diorcinol 3-*O*-α-D-ribofuranoside (**1**), along with seven known compounds, diorcinal (**2**), 3,3′-dihydroxy-5,5′-dimethyldibenzofuran (**3**), cordyol (**4**), gibellulin B (**5**), cyclo-(L-Trp-L-Phe) (**6**), sydonic acid (**7**), and sydowic acid (**8**) (Figure 1B).

## 2. Results and Discussion

Compound **1** was isolated as a colorless viscous oil. A molecular ion was measured at 363.1443 [M + H]^+^ (calcd. for C_19_H_23_O_7_, 363.1444), indicating a molecular formula of C_19_H_22_O_7_ with 9 degrees of unsaturation. The UV spectrum (MeOH) displayed absorption maxima at λ_max_ (log ε) 281 (4.37) and 279 (3.01) nm. The ^1^H- and ^13^C-NMR, DEPT, and HMQC spectroscopic data revealed six olefinic methines, five olefinic quaternary carbons, four oxymethines, an oxymethylene, two methyl groups, and four hydroxyl groups (Table 1). These data suggested that **1** contains a diphenyl ether unit similar to 3,3-dihydroxy-5,5-dimethyl diphenyl ether (diorcinol) [9,10], which was also supported by the HMBC correlations (Table 1). The major difference was the presence of an additional sugar moiety. Signals corresponding to five sugar carbons (100.3, 86.3, 71.5, 69.3, 61.6) are typical for ribofuranosides [11]. The carbohydrate proton (H-1″) appeared as a doublet at δ 5.51 (d, 4.2 Hz) that is confirming the α orientation [11]. The assignment of the sugar signals was further confirmed by careful ^1^H-^1^H COSY, and HMBC analysis (Table 1). The connectivity between α ribofuranose moiety and aglycone was deduced by HMBC correlation from H-1″ to C-3 (δ 158.6). The absolute configuration of the sugar moiety was determined by hydrolysis and GC analysis of compound **1** (experimental section). Thus, **1** was determined as 3′-hydroxy-5,5′-dimethyl-1,1′-diphenyl ether 3-*O*-α-D-ribofuranoside (diorcinol 3-*O*-α-D-ribofuranoside). Based on our knowledge, there were only two diphenyl ether glycosides (cordyols A and B) reported from fungi species and compound **1** was the first diphenyl ether glycoside incorporating D-ribose as a sugar component [12].

Diorcinal (**2**) [13,14], 3,3′-dihydroxy-5,5′-dimethyldibenzofuran (**3**) [15], cordyol (**4**) [12], gibellulin B (**5**) [16], cyclo-(L-Trp-L-Phe) (**6**) [17], sydonic acid (**7**) [18], and sydowic acid (**8**) [19] were known compounds, whose structures were elucidated by comparisons with the literature (MS, ^1^H and ^13^C-NMR data of known compounds are available from supporting information).

**Figure 2:**
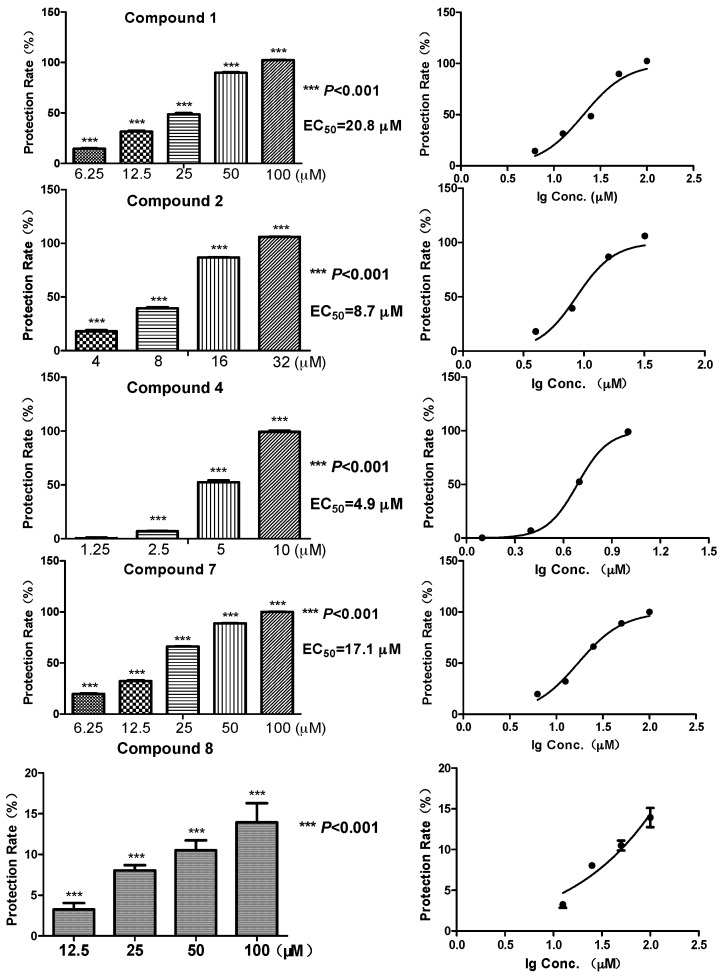
Erythrocyte membrane protection activity (%) of compound **1**, **2**, **4**, **7** and **8** against hemolysis induced by AAPH. The data were expressed as the means ± SD from four individual experiments and were analyzed using a *t*-test to determine any significant differences. *** *p* ≤ 0.001 compared to control group.

Biomembrane protective activity of the representative compounds **1**, **2**, **4**, **7** and **8** (which have enough amounts) was tested using the erythrocytes protective assay [20]. Among the five compounds, the diphenyl ethers **2** and **4** showed a relatively strong protective effect against free radicals, with EC_50_ at 8.7 and 4.9 μM, respectively (Figure 2). While the glycosylated compound **1** showed an activity reduction, with EC_50_ at 20.8 μM. Compound **7**, a benzoic acid with a C_8_ aliphatic side chain, showed moderate activity (EC_50_ at 17.1 μM). In contrast, compound **8**, which had a six-membered ring side chain, showed a significant activity reduction (13.9% membrane protective rate at 100 μM, data not shown in Figure 2). Structures of **7** and **8** suggested the loss of activity might possibly come from the formation of six-membered ring which had a larger steric interference. Meanwhile, the same compounds did not exhibit any radical scavenging activity even at much higher concentrations (200, 400 μM) on DPPH assay, which was carried out at a non-cell condition [20] (Table 2, five concentrations of each compound were examined, data of the highest conctration were shown). Several antioxidative effects of diphenyl ethers were reported before [21,22], whereas the biomembrane protective effects were only illustrated in this research.

**Figure 3:**
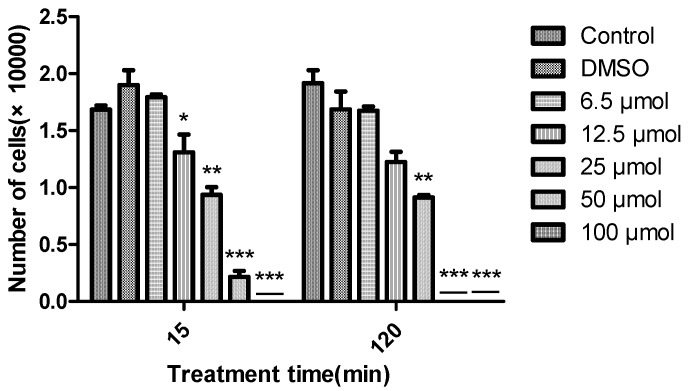
Algicidal effects of diorcinal (**2**) on the growth of *Chattonella marina* after 15 and 120 min of exposure. The data were expressed as the means ± SD from four individual experiments and were analyzed using a *t*-test to determine any significant differences. * *p* ≤ 0.05, ** *p* ≤ 0.01, *** *p* ≤ 0.001 compared to control group.

The algicidal activity of diorcinal (**2**) and sydonic acid (**7**) (which we have enough amounts) against *Chattonella marina*, a bloom forming alga responsible for large scale fish deaths, at different concentrations (6.25, 12.5, 25, 50 and 100 μM) over time (15 and 120 min) were also examined. Greater algicidal activity was observed with higher concentrations and longer treatment times by **2** (Figure 3). **7** did not show any activity even at the highest concentration over the above time period. 15 sulphonyl derivatives of diphenyl ethers were reported as potential algaecides against *Chlorella fusca* and *Anabaena variabilis* [23]. This is the first report about diphenyl esters against *Chattonella marina.*

## 3. Materials and Methods

### 3.1. General Experimental Procedures

Optical rotations were determined on a Jasco P-1020 polarimeter (Jasco, Tokyo, Japan). UV data were recorded on a Perkin Elmer Lambda 25 UV-Vis spectrometer (PerkinElmer, Boston, MA, USA). IR data were recorded using a Nicolet Avatar 330 FT-IR spectrometer (Thermo Scientific, Waltham, MA, USA). NMR spectra were acquired on a Bruker ASCEND 600 MHz NMR magnet system (Bruker, Ettlingen, Germany) using TMS as the internal standard. HR-ESIMS was performed using a BrukermaXis (Bruker, Ettlingen, Germany). Sugar derivatives were measured by an Agilent GCMS, 7890A-5975C system (Agilent Technologies, Santa Clara, CA, USA). Column chromatography was conducted using silica gel (100–200 mesh, Qingdao Marine Chemical Factory, Qingdao, China) and Sephadex LH-20 (Amersham Pharmacia Biotech, Piscataway, NJ, USA). TLC was performed on Merck TLC plates (silica gel 60 RP-18 F_254S_ and silica gel 60 F_254_, Merck Millipore Corporation, Darmstadt, Germany), with compounds visualized by spraying with 5% (*v*/*v*) H_2_SO_4_ in EtOH and then heating on a hot plate. HPLC was performed on a Shimadzu LC-20AT pump equipped with a SPD-20A UV-Vis detector (Shimadzu Corporation, Tokyo, Japan). ZORBAX RX-C_18_ (9.6 × 150 mm I.D. 5 μ), ZORBAX RX- C18 column (4.6 × 250 mm I.D. 5 μ), and a YMC-Pack Pro C18 column (4.6 × 250 mm I.D. 5 μ) were used for semi-preparative and analysis purposes, respectively.

### 3.2. Strain

Fungus SCSIOW3 was isolated from a deep marine sediment sample collected in the South China Sea (111°36.160 E, 17°59.928 N) at a depth of 2134 m. This fungus was characterized as *Aspergillus* sp. based on the analysis of the ITS region sequence with GenebankS1. This fungus was deposited in the Marine Microbial Lab. College of Life Science, Shenzhen University (Shenzhen, China).

### 3.3. Fermentation, Extraction, and Isolation

Both seed and production media have the same constituents (2.0% glucose, 1.0% peptone, 0.5% yeast extract, with the pH adjusted to 7.5). *Aspergillus* sp. SCSIOW3 was cultured in 250 mL Erlenmeyer flasks containing 75 mL of seed medium. After growing at 28 °C, 220 rpm for two days, the cellular material was placed in a sterile Falcon tube and mixed by vortexing for several minutes to create a uniform fungal cell/spore suspension. Aliquots (5 mL) of seed cultures were inoculated into 250 mL of production medium in 1000 mL Erlenmeyer flasks. At the time of inoculation, 0.5 mL aliquots of DMSO-dissolved SBHA and water-dissolved 5-AZA was added in triplicate, resulting in final concentrations in the liquid media of 1 mM SBHA and 1 mM 5-AZA. The same amount of DMSO and water were added to the control group. The resulting cultures were fermented at 28 °C under static conditions for 10 days. The fermented broth of each flask was extracted three times with 250 mL of EtOAc. The EtOAc extracts of each condition were analyzed by reversed-phase HPLC-MS on a ZORBAX RX-C18 (4.6 × 250 mm I.D. 5 μ) eluted with MeOH–H_2_O (0:100–100:0 over 30 min, 1.0 mL/min). For preparative scale up, *Aspergillus* sp. SCSIOW3 was cultivated using 48 bottles of 1000 mL Erlenmeyer flasks containing 250 mL of production medium in the presence of both 1 mM SBHA and 1 mM 5-AZA. The combined EtOAc extract after evaporation (1.5 g) was applied to a Sephadex LH-20 column chromatography with CHCl_3_-MeOH (1:1) to afford five fractions (Fr.1–Fr.5). Fr.4 (336.4 mg) was further isolated by HPLC with a ZORBAX RX-C_18_ (9.6 × 150 mm I.D. 5 μ) by using MeOH–H_2_O (30:80–80:30 over 30 min, 3 mL/min) as eluent to afford six fractions (Fr.4-1–Fr.4-6). Fr.4-3 (82.0 mg) was further purified by HPLC with a YMC-Pack ODS C_18_ column (4.6 × 250 mm I.D. 5 μ) eluted with MeOH–H_2_O (50:50–75:25 over 20 min, 1.0 mL/min) to yield **1** (2.2 mg, t_R_ 18.0 min), Diorcinol (**2**) (24.9 mg, t_R_ 17.8 min), 3,3′-dihydroxy-5,5′-dimethyldibenzofuran (**3**) (1.0 mg, t_R_ 16.3 min), Cordyol (**4**) (2.6 mg, t_R_ 15.4 min). Fr.4-2 (83.2 mg) was further purified by HPLC with a ZORBAX RX-C18 (4.6 × 250 mm I.D. 5 μ) by using MeOH–H_2_O (50:50–60:40 over 15 min, 1.0 mL/min) as solvent to yield gibellulin (**5**) (1.8 mg, t_R_ 12.0 min), Cyclo-(L-Trp-L-Phe) (**6**) (1.7 mg, t_R_ 10.0 min). Fr.4-5 (41.6 mg) was further purified by HPLC with a ZORBAX RX-C18 (4.6 × 250 mm I.D. 5 μ) by using MeOH–H_2_O (60:40–100:0 over 15 min, 1%HAc, 1.0 mL/min) as solvent to yield Sydonic acid (**7**) (23.0 mg, t_R_ 11.5 min), sydowic acid (**8**) (10.0 mg, t_R_ 9.5 min).

Compound **1**: colorless viscous oil, HR-ESI-TOF-MS: *m*/*z* 363.1443 [M + H]^+^, (calcd. for C_19_H_23_O_7_, 363.1444); $[\alpha {]}_{D}^{28}$ = + 59.7° (*c* 0.67, MeOH); UV (MeOH) λ_max_ (log *ε*) 205 (4.21) nm; IR (film) ν_max_: 3391, 2918, 1584, 1462, 1323, 1155, 1038 cm^−1^.

### 3.4. Acid Hydrolysis and GCMS Analysis of Compound **1**

0.5 mg of **1** was hydrolyzed with 1 mL 2N HCl then heated at 80 °C for 3 h under refluxing. The solution was extracted with CHCl_3_ (1 mL × 3) to remove the aglycone. The water layer was neutralized with NaOH (2N), then concentrated to dryness. The dry powders hydrolysis mixture were dissolved in 1 mL of dry pyridine and then added with 1 mL of trimethylsilyl imidazole and heated at 60 °C under stirring for 1 h. After the reaction, an aliquot (0.4 µL) of the supernatant was removed and directly subjected to GC-MS analysis with the other sugar derivatives under the following conditions: Agilent GCMS, 7890A-5975C, Column HP-5MS (30 m × 0.25 mm × 0.25 µm). A temperature gradient system was used for the oven, starting at 100 °C and increasing up to 150 °C at rate 10 °C/min, then increasing up to 260 °C at rate 15 °C/min for 40 min carrier gas N_2_ (1.2 mL/min), injector and detector temp 260 °C, split ratio 1:20. The configurations of the sugar fraction were determined by comparison of the retention times of the corresponding derivative with standard D-ribose at 8.40 min.

### 3.5. Antioxidant Assays

The direct antioxidative activity of the isolated compounds were evaluated by the modified 2,2-diphenyl-1-(2,4,6-trinitrophenyl) hydrazyl (DPPH) radical scavenging assay [13], in which compounds solutions (0.4 M HOAc/NaOAc buffer at 3:1 ratio) were mixed with 20% (*m*/*v*) DPPH ethanol solution, followed with a 30 min incubation in dark and detection at 517 nm. The biomembrane protective assay was tested in erythrocytes which were obtained by centrifuging whole rabbit heart vein blood with 3.2% citrate at 700 g for 10 min at 4 °C. 6 × 10^8^ /mL of the washed erythrocytes were treated with the compounds for 30 min at 37 °C, followed with the addition of 100 mM, 2′-Azobis (2-amidinopropane) dihydrochloride (AAPH) to induce hemolysis for 60 min at 37 °C. The supernatant was measured at 545 nm after a centrifugation at 700 g, 2 min. The biomembrane protective activity was expressed as percentage of a negative control, in which intact erythrocytes in 0.9% saline were measured. Curcumin was used as a positive control.

### 3.6. Algicidal Activity

*Chattonella marina* was cultured in YMNL GXZ Constant temperature illuminate incubator (Nanjing Yimaneli Instrument Equipment Co., Ltd., Nanjing, China).The proliferation of algal was detected through OLYMPUS IX51 inverted microscope (OLYMPUS, Tokyo, Japan). *C. marina* was donated by the Algal Culture Collection of the Institute of Hydrobiology at Jinan University in Guangzhou, China. It was cultivated under a 12:12 h light-dark cycle with 50 μmol photons m^−2^·s^−1^ at 22 °C in a sterilized f/2 medium prepared with natural seawater. Exponential growth phase algal cultures were divided into aliquots for further study. *C. marina* in the exponential growth phase were divided into aliquots and plated in a 12-well plate at a density of 2.9 × 10^4^ cells/well. The tested samples were dissolved in dimethyl sulfoxide (DMSO), and then two-fold serial dilutions were performed. 2 μL of each sample solution was then added in the culture medium to make the final concentrations of 100, 50, 25, 12.5 and 6.5 µM, respectively. 2 μL of DMSO was used as a solvent control. Four parallel repetition was set in each group. The alga was cultured under the conditions described above. Living alga cells in 100 μL culture were counted by a DengXun DSJ-01 counting chamber for zooplankton (Xiamen Den Instrument Co., Ltd., Fujian, China) to detect the proliferation of the algal cells over 15 min and 120 min.

### 3.7 Statistical Analysis

All results were expressed as mean ± SD. Statistical significance (*p* values) of the results was calculated by Student’s *t-*test. The results were considered to be significant when *p* < 0.05 (*), and to be highly significant when *p* < 0.01 (**).

## 4. Conclusions

The present study aimed at activating silent biosynthetic pathways of secondary metabolites and identifying active novel compounds from fungi. In total, 72 marine derived fungi were tested by a chemical epigenetic approach, in which 28 strains (38.8%) showed significant changes on the chemical profiles. Strain *Aspergillus* sp. SCSIOW3 was then scaled up and one new diphenylether-*O*-glycoside (**1**, diorcinol 3-*O*-α-D-ribofuranoside), along with seven known compounds, diorcinal (**2**), 3,3′-dihydroxy-5,5′-dimethyldibenzofuran (**3**), cordyol (**4**), gibellulin B (**5**), cyclo-(L-Trp-L-Phe) (**6**), sydonic acid (**7**), and sydowic acid (**8**), were isolated from the culture treated with a combination of SBHA and 5-AZA. **1** was the first diphenyl ether glycosides incorporating D-ribose as a sugar component. The biomembrane protective effect of erythrocytes and DPPH radical scavenging activities were tested for compounds **1**, **2**, **4**, **7** and **8**. None of these compounds exhibited any radical scavenging activity at high concentrations (200, 400 μM) by DPPH assay. Compounds **2** and **4** exhibited the significant biomembrane protective effect of erythrocytes, which suggested their potential therapeutic use or preventive agents for ROS-associated diseases. In addition, **2** demonstrated algicidal activity against *Chattonella marina*. **7** did not show any algicidal activity.

## Figures and Tables

**Table 1:** NMR spectroscopic data of compound **1** (DMSO-*d*_6_) *^a^*.

Position	1H (mult, *J* in Hz) *^b^*	13C *^c^*	HMBC	^1^H-^1^H COSY
1		157.5 *^d^*		
2	6.42 (s)	104.7	6	4,6
3		158.3		
4	6.63 (s)	112.1	2, 3, 5-CH_3_, 6	2,6
5		140.2		
6	6.44 (s)	112.5	1, 2, 4, 5-CH_3_	2,4
1′		157.5 *^d^*		
2′	6.17 (s)	103.1	1′, 4′, 6′	4′,6′
3′		158.6		
4′	6.35 (s)	111.4	2′, 3′, 6′, 5′-CH_3_	2′,6′
5′		140.3		
6′	6.25 (s)	110.1	1′, 2′, 4′, 5′-CH_3_	2′,4′
1′'	5.51 (d, 4.8)	100.3	3, 3″, 4″	2′'
2′'	4.02 (m)	71.5		1″,3″,2″-OH
3″	3.89 (m)	69.3	1″, 5″	2″,4″,3″-OH
4″	3.94 (m)	86.3		3″,5″
5″	3.48 (m, 2H) *^e^*	61.6	3″, 4″	4″,5″-OH
3′-OH	9.47 s			
2″-OH	4.66 (d, 9.0)			
3″-OH	4.87 (t, 5.4)			
5″-OH	4.81 (d, 5.4)			
5-CH_3_	2.24 (s)	21.3	4, 5, 6	
5′-CH_3_	2.18 (s)	21.2	4′, 5′, 6′	

*^a^* Chemical shifts (δ) in ppm; *^b^* 600 MHz; *^c^* 150 MHz; *^d^* overlapped signal; *^e^* overlapped with water signal.

**Table 2:** DPPH radical scavenging activity (%) of isolated compounds and curcumin (Cur., positive control).

	DPPH Scavenging Activity	
Samples	Conc. (μM)	Rate (%)
Cur.	200	62.4 ± 1.0
1	200	5.2 ± 0.2
2	200	5.8 ± 0.8
4	200	21.3 ± 2.5
7	400	−1.2 ± 1.7
8	400	−0.1 ± 0.8

## Supplementary Materials

The following are available online: Table S1: Fungal strains used in epigenetic experiments. MS, ^1^H- and ^13^C-NMR data of known compounds are also available from supporting information.
