# Supplementary material for: Identification and Biological Evaluation of Secondary Metabolites from Marine Derived Fungi-Aspergillus sp. SCSIOW3, Cultivated in the Presence of Epigenetic Modifying Agents
Source: Molecules. 2017 Aug 4;22(8):1302. doi: 10.3390/molecules22081302 (PMC6152046; doi:10.3390/molecules22081302)
Supplement: Supplementary file 1 [file molecules-22-01302-s001.pdf]

Table S1. Fungal strains used in epigenetic experiments

No.	Strain Lab No.	Strain ID.	Observations[a]
1	TH-01		A
2	TH-03		A
3	TH-06		A
4	TH-07		BD
5	TH-08		A
6	TH-09		A
7	TH-10		A
8	TH-12		BD
9	TH-14		A
10	TH-15		BE
11	TH-16		A
12	TH-17		BD
13	TH-18		A
14	TH-19		BD
15	TH-21		A
16	ML-01		A
17	ML-02		A
18	ML-03		A
19	ML-04		BD
20	ML-05		A
21	ML-07		BD
22	ML-08		A
23	TU-01		A
24	TU-04		BC
25	TU-05		A
26	TU-06		A
27	1a-A2-14		A
28	3a-A2-15		A
29	25e-A3-1		BD
30	B3-11		BC
31	AN	*Aspergillus* nidulans	A
32	W1		A
33	W2	*Aspergillus* sp.	BCE
34	SCSIOW3	*Aspergillus* sp.	BCE (strain in this study)
35	W6	*Fusarium* sp.	BE
36	W7		A
37	W20	*Aspergillus* sp.	BCE
38	W21	*Trichoderma* sp.	BC
39	W26		BC

40	W27	A
41	W28	BC
42	W29	BE
43	F1	A
44	F2	BD
45	F3	A
46	F4	A
47	F5	BCE
48	F6	BD
49	F7	A
50	F8	BE
51	F9	A
52	F10	A
53	F11	BD
54	F12	A
55	F13	A
56	F14	A
57	F15	BD
58	F16	BE
59	F17	BE
60	F18	A
61	F19	A
62	F20	A
63	F21	A
64	F22	A
65	F23	BD
66	F24	A
67	F25	A
68	F26	A
69	F27	A
70	F28	BC
71	F29	A
72	F30	A

[a] Legend to Observations： A: No significant change in the chemical profile. B: Significant change in the chemical profile. C: New signals observed. D: the intensity of signals decreased. E: the intensity of signals increased.

MS, ^{1}H and^{13}C -NMR data of known compounds.

Diorcinal (2): colorless viscous oil, ESI-MS: *m/z* 231.1 $[M+H]^+$, ^{1}H-NMR (DMSO-d_6, 600MHz) δ: 9.45 (1H, s, 3/3'-OH), 6.33 (1H, s, H-2/2'), 6.23 (1H, s, H-4/4'), 6.15 (1H，s，H-6/6'), 2.18 (3H, s, 5/5'-CH_3); ^{13}C-NMR (DMSO-d_6, 150 MHz) δ: 158.5 (C-3/3'), 157.6 (C-1/1'), 140.1 (C-5/5'), 111.2 (C-4/4'), 110.0 (C-6/6'), 102.9 (C-2/2'), 21.1 (5/5'-CH_3);

3,3'-dihydroxy-5,5'-dimethyldibenzofuran (3): colorless viscous oil, ESI-MS: *m/z* 229.2 $[M+H]^+$, ^{1}H-NMR (DMSO-d_6, 600MHz) δ: 9.65 (1H, s, 3/3'-OH); 6.74 (1H, d, J = 2.4 Hz, H-2/2'), 6.55 (1H, d, J = 1.2 Hz H-4/4'), 2.74 (3H, s, 5/5'-CH_3); ^{13}C-NMR (DMSO-d_6, 150 MHz) δ: 157.0 (C-1/1'), 155.7 (C-3/3'), 131.4 (C-5/5'), 115.5 (C-6/6'), 114.0 (C-4/4'), 95.6 (C-2/2') , 24.4 (5/5' -CH_3);

cordyol (4): colorless viscous oil, ESI-MS: *m/z* 269.3 $[M+Na]^+$, ^{1}H-NMR (DMSO-d_6, 600MHz) δ: 6.44 (1H, d, J = 1.2 Hz, H-4'), 6.22 (1H, s, H-4), 6.19 (1H, d, J = 1.2 Hz, H-6'), 6.13 (1H, s, H-6), 6.02 (1H, s, H-2), 2.15 (3H, s, 5-CH_3), 2.10 (3H, s, 5'-CH_3); ^{13}C-NMR (DMSO-d_6, 150 MHz) δ: 159.2 (C-1), 158.2 (C-3), 146.8 (C-3'), 143.2 (C-1'), 139.5 (C-5), 135.3 (C-2'), 127.7 (C-5'), 112.7 (C-6'), 112.6 (C-4'), 109.8 (C-4), 108.0 (C-6), 100.8 (C-2), 21.2 (5-CH_3), 20.5 (5'-CH_3);

gibellulin B (5): colorless viscous oil, ESI-MS: *m/z* 261.1 $[M+H]^+$, ^{1}H-NMR (DMSO-d_6, 600MHz) δ 9.43 (1H, s, 5'-OH), 8.85 (1H, s, 3-OH), 8.54 (1H, s, 2-OH), 6.34 (1H, d, J = 1.2 Hz, H-4'), 6.22 (1H, d, J = 1.2 Hz, H-2'), 6.21 (1H, s, H-4), 2.12 (3H, s, 3'-CH_3), 2.05 (3H, s, 5-CH_3); ^{13}C-NMR (DMSO-d_6, 150 MHz) δ: 145.6 (C-5'), 142.1 (C-1'), 142.0 (C-2), 133.4 (C-5), 132.2 (C-3'), 131.8 (C-1), 131.7 (C-3), 128.7

(C-6'), 114.2 (C-6), 112.6 (C-4'), 110.9 (C-4), 107.6 (C-2'), 21.0 (3'-CH_3), 14.8 (5-CH_3);

cyclo-(L-Trp-L-Phe) (6): white wax, ESI-MS: *m/z* $[M+Na]^+$ 356.2, ^{1}H-NMR (DMSO-d_6, 600MHz) δ: 10.93 (1H, s, H-1), 7.92 (1H, d, *J* = 1.8 Hz, H-12), 7.72 (1H, s, H-15), 7.48 (1H, d, *J* = 8.4 Hz, H-4), 7.32 (1H, d, *J* = 7.8 Hz, H-7), 7.19 (2H, m, H-20, 22), 7.16 (1H, m, H-21), 7.07 (1H, t, H-6), 6.98 (1H, t, H-5), 6.96 (1H, d, *J* = 1.8 Hz, H-2), 6.70 (2H, d, *J* = 6.6 Hz, H-19, 23), 3.97 (1H, s, H-11), 3.85 (1H, s, H-14), 2.53 (1H, dd, *J* = 12.0, 5.5Hz, H-10), 2.80 (1H, dd, *J* = 12.0, 4.4 Hz, H-10), 2.45 (1H, m, H-17), 1.83(1H, m, H17); ^{13}C-NMR (DMSO-d_6, 150 MHz) δ: 166.9 (C-16), 166.3(C-13), 136.6 (C-18), 136.1 (C-8), 129.8 (C-19, 23), 128.1 (C-20, 22), 127.6 (C-3), 126.4 (C-21), 124.5 (C-2), 121.0 (C-6), 118.8 (C-4), 118.5 (C-5), 111.4 (C-7), 108.8 (C-9), 55.7 (C-14), 55.3 (C-11), 39.9 (C-17), 29.7(C-10);

sydonic acid (7): colorless viscous oil, ESI-MS *m/z* 289.2 $[M+Na]^+$, ^{1}H-NMR (DMSO-d_6, 600MHz) δ: 7.41 (1H, d, *J* = 8.4 Hz, H-5), 7.33 (1H, dd, *J* = 7.8, 1.8 Hz, H-4), 7.29 (1H, d, *J* = 1.8 Hz, H-2), 1.96 (1H, m, H-8b), 1.65 (1H, m, H-8a), 1.50 (3H, s, 7-CH_3), 1.42 (1H, m, H-11), 1.26 (1H, m, H-9b), 1.05 (2H, m, H-10), 0.97 (1H, m, H-9a), 0.77 (3H, d, *J* = 3.6 Hz, H-13), 0.76 (3H, d, *J* = 2.4 Hz, H-12); ^{13}C-NMR (DMSO-d_6, 150 MHz) δ: 167.3 (s, 3-COOH), 154.5 (s, C-1), 137.9 (s, C-6), 130.0 (s, C-3), 127.0 (d, C-5), 119.7 (d, C-4), 116.6 (d, C-2), 74.6 (s, C-7), 41.2 (t, C-8), 38.8 (t, C-10), 28.3 (q, 7-CH_3), 27.3 (d, C-11), 22.6 (q, C-13), 22.4 (q, C-12), 21.3 (t, C-9);

sydowic acid (8): white wax, ESI-MS *m/z* 263.3 $[M-H]^-$, ^{1}H-NMR (DMSO-d_6, 600MHz) δ: 9.36 (1H, s, H-15), 7.38 (1H, dd, *J* = 8.0, 1.5 Hz, H-4), 7.33 (1H, d, *J* =

8.0 Hz, H-5), 7.27 (1H, d, J = 1.5 Hz, H-2), 2.24 (1H, m, H-8b), 1.79 (1H, m, H-8a), 1.69 (1H, m, H-9b), 1.62 (1H, m, H-9a), 1.48 (2H, m, H-10), 1.43 (3H, s, H-14), 1.25 (3H, s, H-13), 0.95 (3H, s, H-12); ^{13}C-NMR (DMSO-d_6, 150MHz) δ: 167.1 (C-15), 155.6 (C-1), 137.0 (C-6), 130.7 (C-3), 125.6 (C-5), 120.3 (C-4), 117.2 (C-2), 76.6 (C-7), 73.9 (C- 11), 36.0 (C-10), 33.2 (C-8), 30.7 (C-13), 29.8 (C-14), 26.7 (C-12), 16.1 (C-9);
